# Diptheria

**Published:** 1867-09

**Authors:** Reuben Searcy

**Affiliations:** Tuscaloosa, Ala.


### ARTICLE III.

*Diphtheria.* By Reuben Searcy, M. D., Tuscaloosa, Ala.

There being such a diversity of opinion in the treatment of Diphtheria, I have concluded to give the result of my experience. No doubt there is a specific morbid poison that has entered the system, and located on the tonsils and throat, but in what way I cannot tell, or from what cause it has originated, is equally obscure; consequently, the treatment is experimental.

At first, I adopted the plan recommended, of cauterizing the ulcers in the tonsils with the nitrate of silver, opening the bowels with aperients, gargling the throat with red pepper tea, seasoned with salt, and stimulating applications to the upper part of the neck, externally. Some got well, others died; the disease assuming croupy symptoms, and leading to the formation of false membrane. In one instance I performed the operation of tracheotomy, which prolonged the suffering of the child several hours. I do not remember of relieving a case after the formation of the false membrane, although I used hot vapor inhalations to dissolve the membrane, and emetics to dislodge it, together with the application of kerosene externally, hoping, by its blistering properties, to translate the inflammation to the outside surface—all to no purpose. I afterwards succeeded better in curing the disease with muriated tincture of iron. I now treat the disease with a saturated solution of the chlorate of potash, acidulated with muratic aci; i. e. one hundred grains of the chlorate of potash, into four ounces of water, adding one drachm of muriatic acid. To a child ten years old, give a teaspoonful every two hours, in water or sweetened water, and less to younger children. I directed two pieces of fat bacon to be sewed to a piece of cloth and bound to the neck over the tonsils, and to be worn until convalescence. Also, when the skin of the body is hot and dry, to rub the patient all over with a piece of bacon rind, then wash off with warm water and soap. This always lessens fever, producing sleep and perspiration. This should be repeated as often as the hot dry skin requires. Gentle aperients, gruels, teas, &c., should be directed, but an active purgative or emetic should never be used. I have been uniformily successful, for one year and a half, since adopting the above practice.

